# Feasibility and long-term safety of Ho:YAG laser lithotripsy in broncholithiasis patients

**DOI:** 10.1186/s12890-021-01407-8

**Published:** 2021-03-10

**Authors:** Yuan Cheng, Guangfa Wang, Wei Zhang, Hong Zhang, Xi Wang

**Affiliations:** Department of Respiratory and Critical Care Medicine, Peking University First Hospital, 8 Xi Shi Ku Street, Xi Cheng District, Beijing, 100034 China

**Keywords:** Ho:yag laser, Lithotripsy, Broncholithiasis

## Abstract

**Background:**

Treatment of broncholithiasis is complex, especially in the case of a large or transbronchial broncholith. Holmium-yttrium aluminum garnet (Ho:YAG) laser lithotripsy may be a useful treatment in broncholithiasis; however, as it is not yet common practice, the optimal parameters are unknown.

**Methods:**

We performed a single-center retrospective analysis of the clinical data of 13 broncholithiasis patients who underwent Ho:YAG laser lithotripsy from May 2012 to October 2018.

**Results:**

For the 13 patients (2 males and 11 females), Ho:YAG laser lithotripsy was performed 17 times, in total. All procedures were performed under general anesthesia with rigid bronchoscopy. We initially set the Ho:YAG laser to a pulse frequency of 5 Hz and a pulse energy of 0.8 J, gradually increasing these as required. The pulse frequency range we employed was 5–15 Hz, and the pulse energy range was 0.8–1.6 J. All broncholiths were successfully extracted after lithotripsy, and all symptoms improved. Hemoptysis, bronchial esophageal fistula, and pneumonia were the most common complications; however, there were no long-term complications.

**Conclusions:**

Ho:YAG laser lithotripsy is an effective and safe treatment for broncholithiasis, over a long-term follow up.

## Background

Broncholithiasis is a rare disease that is caused by granulomatous infection such as tuberculosis, histoplasmosis, actinomycosis, and nocardiosis [1, 2]. The necessity of treatment depends on the symptoms due to airway obstruction such as cough, hemoptysis, dyspnea, or recurrent pneumonia. The most common methods of treatment are therapeutic bronchoscopy and surgery. Most reports of therapeutic bronchoscopy indicate that broncholiths are treated as foreign bodies, removed using foreign body forceps, rigid forceps, or a Fogarty balloon catheter [3]. Jin et al. [4] reported a treatment of intraluminal broncholith with a success rate of only 50%(2/4), Seventeen out of 19 patients with transluminal broncholiths received thoracotomy, only two patients received rigid bronchoscope. Therapeutic bronchoscopy had a low success rate especially for transluminal broncholiths. Thoracotomy had a better success rate, but more postoperative complications, higher mortality rate and loss of lung function. Bronchoscopic treatment may be difficult in the cases of large or transbronchial broncholiths. In such cases, broncholiths may first be fragmented by laser lithotripsy [5–7]; There are several case reports of continuous wave neodymium-YAG(Nd:YAG) laser for broncholith lithotripsy [5, 6]. But for lithotripsy of the airway, Nd:YAG lasers generate too much heat, and may cause collateral damage to the surrounding normal tissue. McCaughan JS et al. [8], and Ferguson JS et al. [5] reported use of Ho:YAG lithotripsy in three broncholithiasis patient but less experience with all types of broncholiths, appropriate laser parameters, procedure protocol and complications. However, as it is not yet common practice, the appropriate type of laser, and the optimal parameters remain unclear. With our experience of lithotripsy in the treatment of urinary calculi, we performed holmium-yttrium aluminum garnet (Ho:YAG) laser lithotripsy in broncholithiasis patients, as reported below.

## Methods

### Patients

We performed a retrospective analysis of patients who underwent Ho:YAG laser lithotripsy from May 2012 to October 2018 in the Department of Respiratory and Critical Care Medicine, Peking University First Hospital. The ethics committee of Peking University First Hospital approved this study.

### Equipments

1. Rigid bronchoscope: (KARL STORZ SE & Co. KG, Tuttlingen, Germany).
2. Flexible bronchoscope: BF-260 or BF-290 series (Olympus Corporation, Tokyo, Japan).
3. Manual jet ventilation system: Manujet III (VMB Medizintechnik GmbH, Sulz am Neckar, Germany).
4. Ho:YAG laser: Lumenis laser system (output range: pulse frequency 5–40 Hz, pulse energy 0.2–3.5 J; Lumenis Ltd., Yokne’am, Israel) or Jiangsu Wuxi Dahua DHL-1 series laser system (output range: pulse frequency 1–50 Hz, pulse energy 0.1–5.0 J; Wuxi Dahua Laser Equipment Co., Ltd., Wuxi, China).
5. Optical fiber: SlimLine EZ 365μ (Boston Scientific Corporation, Marlborough, MA, USA) or Scopesafe fiber 365 μm (Mayumana Healthcare B.V., Hillegom, The Netherlands).

### Laser protocol

We initially set the Ho:YAG laser to a pulse frequency of 5 Hz, and a pulse energy of 0.8 J; these were adjusted as required throughout each procedure. The optical fiber was inserted through the working channel of either a rigid or a flexible bronchoscope.

### Procedure protocol

An attempt was made to fill the bronchus with saline, and a clear view was required before the laser was activated.
Where the bronchus could not be filled with saline, the tip of the optical fiber was placed in direct contact with the surface of the broncholith. The pulse duration was carefully controlled to reduce the risk of overheating the broncholith, and saline was repeatedly used for cooling after several pulses.
Flexible bronchoscope be may use through rigid bronchoscope working channel according to the location of broncholiths.
(1) For large broncholiths, the optical fiber tip was placed into direct contact with the broncholith, and a hole was drilled into its core. Thereafter, the optical fiber tip was placed in the core of the broncholith, and the laser was used to fragment the broncholith from the inside. (2) For small broncholiths, the optical fiber tip was placed 1–2 mm from the surface of the broncholith, from where the shock wave of the laser fragmented the broncholith.

The frequency range employed was 5–15 Hz, and the energy range employed was 0.8–1.6 J. Each pulse was maintained for less than 2 s. After fragmentation, broncholiths were extracted by suction or with forceps.

### Follow-up

All patients in the study were followed up for at least one year. Symptoms that could be ascribed to broncholiths were recorded, followed by a chest computed tomography (CT) scan or bronchoscopy, where necessary.

## Results

We reviewed 13 patients in this study. Of these, 2 were male and 11 were female; patients’ age ranged from 46 to 76 years (median = 59 years). In total, we performed the procedure 17 times for these patients. All but one of the patients presented with one or more of the following symptoms: wheezing, coughing, pneumonia, or recurrent pneumonia. The remaining patient presented with only atelectasis. All patients had mediastinal, pulmonary, or pleural calcification in addition to the broncholiths, as visualized with chest CT. Acid-fast stain testing was positive only for patient 7, and interferon-γ release assays were positive for all patients (Table 1).

**Table 1:** Characteristics of the patients

Pt No	Symptom	Broncholith Location	Classification	AFB	IGRA
1	Cough	Right intermediate bronchus	Transbronchial	Negative	Positive
2	Cough	Right main bronchus	Transbronchial	Negative	Positive
3	Recurrent pneumonia	Right middle bronchus	Endobronchial	Negative	Positive
4	Hemoptysis	Right B8	Endobronchial	Negative	Positive
5	Recurrent pneumonia	Right main bronchus	Transbronchial	Negative	Positive
6	Cough	Left lower lobe	Transbronchial	Negative	Positive
7	Pneumonia	Left lingula bronchus	Transbronchial	Negative	Positive
8	Cough	Right intermediate bronchus	Transbronchial	Positive	Positive
9	cough	Right middle bronchus	Endobronchial	Negative	Positive
10	Wheeze	Right main bronchus	Transbronchial	Negative	Positive
11	Wheeze	Right intermediate bronchus	Transbronchial	Negative	Positive
12	Wheeze	Left main bronchus	Endobronchial	Negative	Positive
13	No symptom	Right lower lobe	Endobronchial	Negative	Positive

*BEF* bronchoesophageal fisula, *AFB* acid-fast stain testing, *IGRA* interferon-γ release assays

All procedures were performed under general anesthesia and using rigid bronchoscopy. Broncholiths were hard, irregular, and yellow or yellowish white, and several were covered with granulation tissue. For eight patients, the broncholiths were transbronchial, and for five patients they were endobronchial. All attempts under bronchoscope failed including forceps, rigid forceps and snares to remove broncholiths before laser treatment, because broncholiths were too large or embedding into bronchial lumen.

**Fig. 1:**
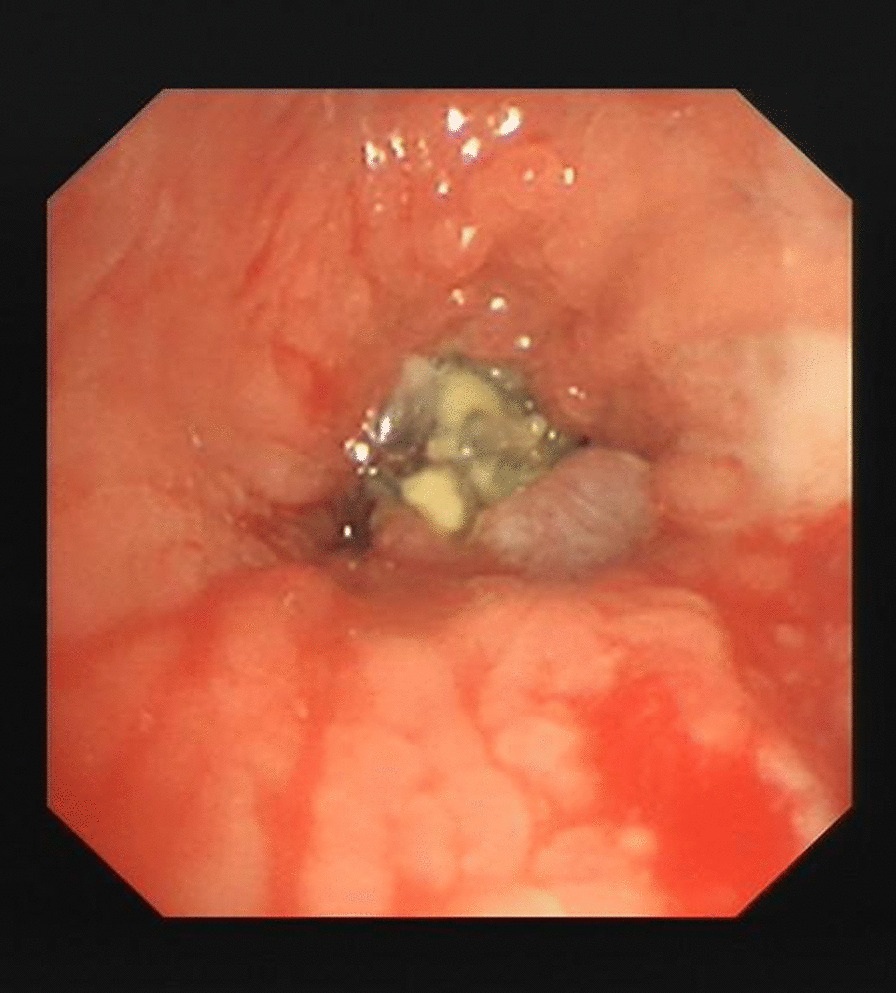
A large broncholith in the right intermediate bronchus (patient 11, before procedure)

**Fig. 2:**
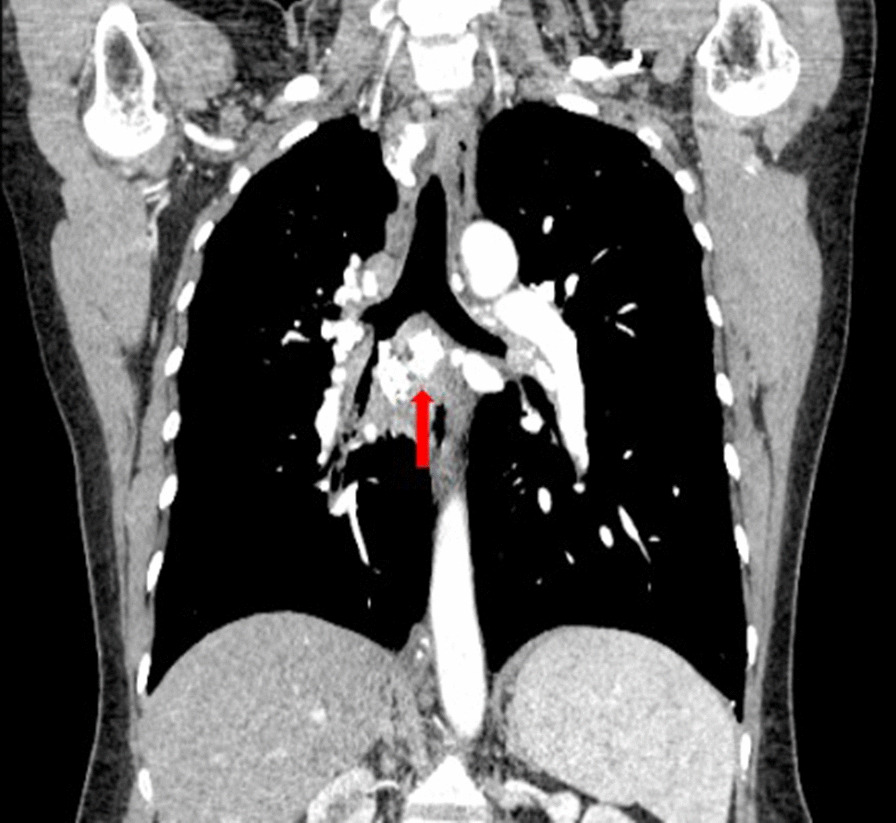
Chest CT scans with contrast show large transbronchial broncholith in the right intermediate bronchus (red arrow) (patient 11, before procedure)

**Fig. 3:**
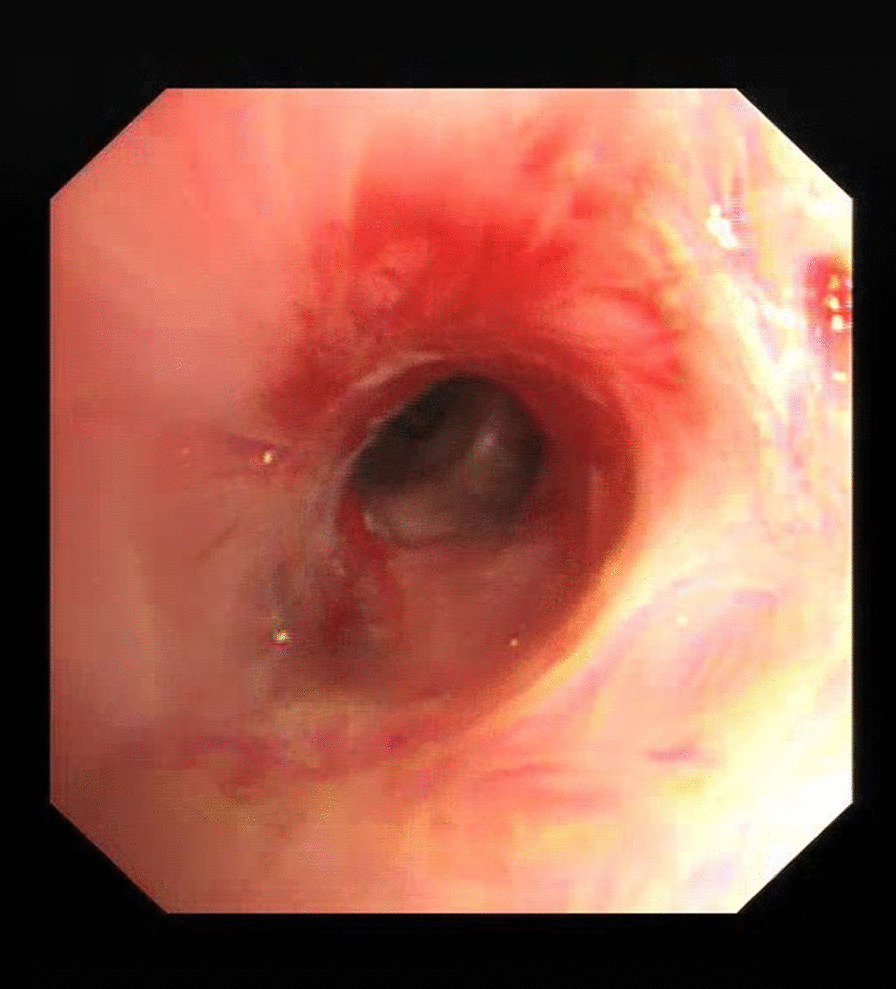
The right intermediate bronchus reopen, with no residual broncholith (patient 11, after five times procedures)

**Fig. 4:**
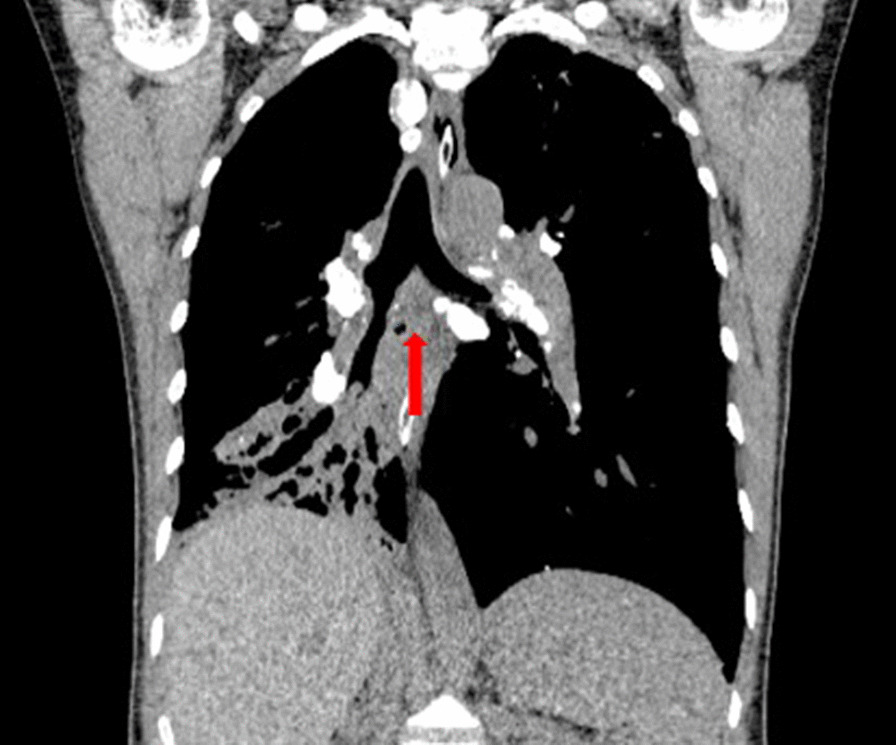
Chest CT scans shows no residual broncholith in the previous location (red arrow) (patient 11, after five times procedures)

Patient 11 had a large broncholith in the right intermediate bronchus (Figs. 1 and 2), and underwent the procedure five times over half a year. Thereafter, there was no stenosis in the right intermediate bronchus (Fig. 3), and chest CT scan revealed no residual broncholiths (Fig. 4). Median procedure duration was 100 min. Median procedure time for transbronchial and endobronchial broncholiths was 102 min and 67 min, respectively (Table 2). All broncholiths were successfully extracted after lithotripsy.

**Table 2:** Laser parameters, complications and follow-up

Pt. no.	Laser energy (J)	Laser frequency	procedure time (minutes)	Perioperative complications	Follow-up
1	1	5–10	160	BEF	BEF recovered after 3 months, survived
2	0.8–1	5	149	Life-threatening hemoptysis	Survived
3	0.8–1	5–8	67	Pneumonia	Survived
4	1	10	15	Pneumonia	Survived
5	1.2	10	104	None	Survived
6	0.8	10	100	None	Survived
7	0.8	8	63	None	Survived
8	0.8	8–10	37	None	Survived
9	1	10	116	None	Survived
10	1–1.2	10	22	Acute asthma attack	Survived
11	0.8–1.2	8–10	129	None	BEF recovered after 4 months, survived
	0.8–1.2	10	112	None	
	0.8–1.2	8–10	54	None	
	0.8–1.6	8–10	142	None	
	1–1.6	10–15	75	BEF	
12	0.8	8	33	None	Survived
13	0.8–1.2	8–10	135	None	Survived

Several patients experienced complications:

1. Life-threatening hemoptysis: we defined life-threatening hemoptysis as hemoptysis more than 200 ml/h [9], or caused oxygen saturation < 90%, persisting for more than 1 min during bronchoscope procedures. One patient experienced such life-threatening hemoptysis (Patient 2). We hypothesized that it was caused by bronchial artery damage. Therefore, we isolated the right airway with a rigid bronchoscope, and achieved hemostasis using gauze, gelatin compressed sponge, and rigid forceps. Double-lumen endotracheal intubation and single-lung ventilation was initiated, and the patient was transferred to the intensive care unit. On the sixth day after the procedure, the patient was successfully extubated.
2. Bronchoesophageal fistula (BEF): two patients (patient 1 and 11) had a bronchial esophageal fistula after the procedure. Both had a transbronchial broncholith located in the right intermediate bronchus, which was completely removed. Patient 1 expectorated a bean-sized stone after the procedure. For patient 11, the whole body of the broncholith was removed during the last procedure. These patients were diagnosed with BEF via upper gastroenterography and upper endoscopy. Both patients’ fistula was located at the original site of the broncholith. They received enteral nutrition and recovered from BEF after several months.
3. Two patients had fever after the procedure, which improved after antibiotic treatment. One patient had an acute asthma attack, which improved after steroid treatment.

There was no airway fire or damage to the bronchoscope.

Prognosis: all patients were followed up until September 2019, with a median follow-up time of 3 years (range 1–7 years). All patients survived with no clinical symptoms at the final follow-up. The patients with BEF fully recovered within 3–4 months. All patient survived, and there were no long-term complications and no disease recurrence.

## Discussion

In many countries, including China, reported tuberculosis rates are twice as high in men as in women [10, 11]. Mediastinal tuberculous lymphadenitis, however, has been reported to be more common in females [12, 13], although the reasons are not well understood. Mediastinal lymphadenitis caused by tuberculosis and histoplasmosis are the most common etiologies of broncholithiasis. All patients in the present study had a history of tuberculosis with old pulmonary tuberculosis lesions. Positive interferon-γ release assays may indicate latent tuberculosis infection in these patients. We believed old or latent pulmonary tuberculosis infection was a possible cause of these broncholithiasis patients. As the position of lesions relative to the airway can be difficult to determine by chest CT, follow-up bronchoscopy should be performed to analyze calcification lesions detected near or in the airway. In addition, broncholiths of three patients in the present study were covered by granulomas. As granulomas may mimic bronchial carcinoma, a biopsy is often necessary to rule out neoplasm. Broncholithiasis is generally classified into three categories depending on whether the broncholiths are purely endobronchial, peribronchial, or mixed (transbronchial) [2]. This classification is important, as it may have an impact on the therapeutic approach and potential complications.

As the absorption maximum of water (1940 nm) is close to the wavelength of the Ho:YAG laser, it can absorb much more Ho:YAG laser energy than that of a Nd:YAG laser (wavelength: 1064 nm). Furthermore, a Ho:YAG laser has a lower penetration depth (only 0.4 mm) in human tissues than does a Nd:YAG laser, which limits the risk of collateral damage. A Ho:YAG laser delivers short pulses of optical energy to the tip of the optical fiber, which causes instantaneous saline evaporation and creates a cavitation bubble; this is known as the “Moses effect.” This cavitation bubble oscillates and violently implodes, generating a shockwave that is transmitted to the target broncholith, causing its fragmentation. For maximum fragmentation, the optimal distance between the fiber tip and the broncholith is 1–2 mm [14, 15].

There are four main points that we want to highlight:

1. Laser parameters: choosing the appropriate parameters can increase the efficiency of lithotripsy and reduce the risk of complications. In vitro experiments have revealed that, for the same output power, low frequency-high pulse energy yields the highest efficiency of lithotripsy [16]. We started our procedures with a pulse frequency of 5 Hz and a pulse energy 0.8 J. The highest pulse frequency we used was 15 Hz, and the highest pulse energy we used was 1.6 J. Within these parameters, we have demonstrated its safety and efficiency. We do not recommend using a high pulse frequency. However, if is clearly insufficient, the pulse energy may be appropriately increased.
2. Lithotripsy technique: The energy from a Ho:YAG laser results in a cavitation bubble with a diameter of 2 mm. If the tip of the optical fiber is too close to the surface of the broncholith (< 1 mm), it results in non-optimal cavitation bubble formation, causing a “drilling effect” rather than fragmentation. The distance between the tip of the optical fiber and the broncholith should be 1–2 mm for optimal fragmentation. If the distance is too large, the shock wave will attenuate before it reaches the broncholith.
3. Strategy for different types of broncholiths: Chest CT scans with contrast should be reviewed carefully before lithotripsy, particularly for the position of the broncholith in the mediastinum (e.g., involvement of arteries or the esophagus), and the type of broncholith. (1) Endobronchial broncholiths can be fragmented via lithotripsy, and all fragments should be extracted, unless there is obvious artery involvement. (2) For transbronchial broncholiths, the endoluminal section is the focus, as excessive treatment of the peribronchial section may cause massive hemoptysis or fistula.
4. Safety and complications: (1) The shell of an optic fiber is fragile and flammable, and its integrity should be verified before and during the procedure. (2) Lithotripsy should be performed under general anesthesia, in combination with rigid or flexible bronchoscopy, due to the risk of life-threatening hemoptysis. (3) During laser activation, the fraction of inspired oxygen should be lower than 40%, and jet ventilation should be halted. (4) The fiber should be held at a distance of at least 4 mm from the distal end of the scope, to protect the bronchoscope. (5) The bronchus must be filled with saline and the optical fiber should be close to or in contact with the broncholith. (6) Hemoptysis is one of the most common causes of fatality due to lithotripsy. Transbronchial broncholiths in the right intermediate bronchus and in the right main bronchus are more likely to result in massive hemoptysis or fistula. If life-threatening hemoptysis does occur, a bronchial blocker, a double-lumen endotracheal tube, bronchial artery embolization, or lobectomy should be implemented. (7) If a broncholith is located within a sinus fistula, the fistula will be exposed upon lithotripsy. We observed such BEF in two patients (patient 1 and 11) in our present study. (8) Following lithotripsy, obstructive pneumonia may result from mucosal edema and necrosis. We observed this phenomenon in two patients in the current study: they had postoperative fever, and pneumonia was confirmed via computed radiography or CT. The pneumonia may be improved with antibiotic treatment. (9) Overheating is a possibility during such procedures, and may damage the bronchoscope. Airway fire is another possible complication, the risk of which may be reduced by filling the bronchus with saline during laser activation, as we did in the current study. (10) Broncholithiasis may recur in the case of transbronchial broncholiths, due to broncholith movement over time. The intact section of the broncholith may protrude into the bronchial tree over the months following the procedure, and patients may require multiple procedures (e.g., in the case of patient 11). Loose broncholiths may also be expectorated after lithotripsy.

## Conclusions

In summary, we demonstrated the safety and efficiency of Ho:YAG laser lithotripsy treatment of patients with broncholithiasis, when using a low pulse frequency and rigid or flexible bronchoscopy. Its energy is readily absorbed by water and its penetration is weak, reducing damage to normal tissue. Transbronchial Ho:YAG laser lithotripsy has a high treatment success rate, which is a promising treatment for broncholithiasis; however, further research is required, preferably multicenter studies with larger sample sizes, in order to verify our findings.

## Data Availability

The datasets used and/or analysed during the current study available from the corresponding author on reasonable request.

## Abbreviations

Ho:YAG: Holmium-yttrium aluminum garnet
Nd:YAG: Neodymium-YAG
CT: Computed tomography
BEF: Bronchoesophageal fistula

## References

[1] Lee T, Woods C, O'Hagan A (2013). Broncholithiasis from histoplasmosis in a pediatric patient: case reports and review of literature. J. Pediatric Infect Dis Soc.

[2] Alshabani K, Ghosh S, Arrossi AV, Mehta AC (2019). Broncholithiasis—a review. Chest.

[3] Krishnan S, Kniese CM, Mankins M, Heitkamp DE, Sheski FD, Kesler KA. Management of broncholithiasis. J. Thorac. Dis. 2018;1(1). 10.21037/jtd.2018.07.15 PMC6218369 30505529

[4] Jin Y-X, Jiang G-N, Jiang L, Ding J-A (2014). Diagnosis and treatment evaluation of 48 cases of broncholithiasis. Thorac. Cardiovasc. Surg..

[5] Ferguson JS, Rippentrop JM, Fallon B, Ross AF, McLennan G (2006). Management of obstructing pulmonary broncholithiasis with three-dimensional imaging and holmium laser lithotripsy. Chest.

[6] Miks VM, Kvale PA, Riddle JM, Lewis JW (1986). Broncholith removal using the YAG laser. Chest.

[7] Snyder RW, Unger M, Sawicki RW (1998). Bilateral partial bronchial obstruction due to broncholithiasis treated with laser therapy. Chest.

[8] McCaughan JS, Heinzmann HG, McMahon D (1996). Impacted broncholiths removed with the holmium: YAG laser. Lasers Surg Med.

[9] Agmy GM, Wafy SM, Mohamed SAA, Gad YA, Mustafa H, El-Aziz A (2013). Bronchial and nonbronchial systemic artery embolization in management of hemoptysis: experience with 348 patients. ISRN Vasc Med.

[10] Chen M, Kwaku A, Chen Y, Huang X, Tan H, Wen S (2014). Gender and regional disparities of tuberculosis in Hunan, China. Int. J. Equity Health.

[11] Schurz H, Salie M, Tromp G, Hoal EG, Kinnear CJ, Möller M (2019). The X chromosome and sex-specific effects in infectious disease susceptibility. Hum. Genomics.

[12] Fontanilla J-M, Barnes A, von Reyn FC (2011). Current diagnosis and management of peripheral tuberculous lymphadenitis. Clin Infect Dis.

[13] Mekonnen D, Derbie A, Abeje A, Shumet A, Nibret E, Biadglegne F, Munshae A, Bobosha K, Wassie L, Berg S (2019). Epidemiology of tuberculous lymphadenitis in Africa: a systematic review and meta-analysis. PLoS ONE.

[14] Patel SN, Rosenkranz L, Hooks B, Tarnasky PR, Raijman I, Fishman DS, Sauer BG, Kahaleh M (2014). Holmium-yttrium aluminum garnet laser lithotripsy in the treatment of biliary calculi using single-operator cholangioscopy: a multicenter experience (with video). Gastrointest Endosc.

[15] Ventimiglia E, Doizi S, Kovalenko A, Andreeva V, Traxer O. Effect of temporal pulse shape on urinary stone phantom retropulsion rate and ablation efficiency using holmium:YAG and super-pulse thulium fibre lasers. BJU Int. 2020. 10.1111/bju.15079 32277557

[16] Kronenberg P, Traxer O (2014). In vitro fragmentation efficiency of holmium: yttrium-aluminum-garnet (YAG) laser lithotripsy–a comprehensive study encompassing different frequencies, pulse energies, total power levels and laser fibre diameters. BJU Int..

